# A Pro-Inflammatory Diet Is Associated With an Increased Odds of Depression Symptoms Among Iranian Female Adolescents: A Cross-Sectional Study

**DOI:** 10.3389/fpsyt.2018.00400

**Published:** 2018-08-29

**Authors:** Nitin Shivappa, James R. Hebert, Asal Neshatbini Tehrani, Bita Bayzai, Farah Naja, Bahram Rashidkhani

**Affiliations:** ^1^Cancer Prevention and Control Program, University of South Carolina, Columbia, SC, United States; ^2^Department of Epidemiology and Biostatistics, Arnold School of Public Health, University of South Carolina, Columbia, SC, United States; ^3^Department of Community Nutrition, Faculty of Nutrition Sciences and Food Technology, National Nutrition and Food Technology Research Institute (WHO Collaborating Center), Shahid Beheshti University of Medical Sciences, Tehran, Iran; ^4^Nutrition and Food Sciences Department, American University of Beirut, Beirut, Lebanon

**Keywords:** dietary inflammatory index, diet, inflammation, depression, Iran

## Abstract

**Background:** The relation between dietary inflammation and risk of depression has not been widely explored. We examined the association between the inflammatory effect of the diet and the odds of depression among Iranian female adolescents.

**Methods:** Using a stratified cluster sampling technique, 300 female adolescents aged 15–18 years were recruited from schools in Tehran between years 2014–2015. Depression was assessed using the Depression, Anxiety and Stress Scale (DASS)- a 21-point scale. The dietary inflammatory index (DII®) was used to evaluate the inflammatory potential of the diet. Dietary intake was assessed using a validated food frequency questionnaire. In addition to descriptive statistics, multivariable linear and logistic regression were used to calculate confounder-adjusted beta estimates and odds ratios.

**Results:** In total, 88 females (30%) had at least a moderate level of depressive symptoms (DASS > 6). Females with the most pro-inflammatory diet had higher DASS depression score (β = 1.67; 95% CI = 0.03, 3.31) and were at 3.96 (95% CI = 1.12, 13.97) times higher odds of having at least moderate depressive symptoms, compared to females with the least anti-inflammatory diets.

**Conclusion:** These data suggest that Iranian adolescent females eating a pro-inflammatory diet, as indicated by higher DII scores, had greater odds of having at least moderate depressive symptoms.

## Introduction

Depression is expected to become the world's second leading disease burden, after cardiovascular disease, by 2020 according the World Health Organization (1). Women are more than twice as likely to be diagnosed with depression compared with men (2). In Iran, major depression is the most prevalent mood disorder, with a prevalence rate of 2.98% among the entire population and 4.38% among women (3). The peak prevalence is between 25 and 44 years of age; however, recent data indicate that depression is occurring at younger ages (4, 5). Several metabolic and inflammatory processes, such as reduced insulin sensitivity, elevations in plasma homocysteine levels and, perhaps more importantly, increased production of pro-inflammatory cytokines and endothelial dysfunction, seem to be the major factors responsible for the depression (3, 4). Proinflammatory cytokines like c-reactive protein and interleukin-6 act by reducing brain monoamine levels, activating neuroendocrine responses, promoting excitotoxicity (increased glutamate levels), and impairing brain plasticity modulate mood behavior which can result in depression (6). Various dietary components have different effects on inflammation (7–9). A prudent dietary pattern high in fish, yogurt, pulses, rice, fruit, vegetables, and pasta has been shown to be associated with lower concentrations of intermediary inflammatory markers (10).

Various studies have been conducted to evaluate dietary exposures in relation to depression (11–15). In general, prospective cohort studies have shown that dietary patterns rich in anti-inflammatory components such as fruits, vegetables, olive oil, and legumes may be protective against depression (16–19). By contrast, increased risk has been observed with “pro-inflammatory” dietary patterns rich in saturated fat, omega 6 fatty acids, and refined carbohydrates (20, 21). A literature-derived, population-based dietary inflammatory index (DII) was developed to assess the inflammatory potential of an individual's diet (22). DII has previously been shown to be associated with various inflammatory markers in different populations (23–28) including among Iranians (29, 30). With respect to health outcomes, DII was significantly associated with different health outcomes ranging from cardiovascular diseases (31–33), cancer (34–37), overall and disease-specific mortality (38–42) to various mental health disorders (43–47). Previously, DII has been shown to be associated with depression in various studies conducted in Western population (48–53) but no study has been conducted in Iran whose dietary habits and culture are very different from Western populations. Our aim was to assess the association between the inflammatory potential of diet of adolescent Iranian females, as indicated by higher DII scores, and dimensions of depression as measured by Depression Anxiety Stress Scale-21 (DASS-21) (54).

## Methods and materials

### Study population

This cross-sectional study of 300 adolescents females aged 15–18 years was carried out in Tehran (capital of Iran) from 2014 to 2015. Participants were randomly chosen by stratified cluster sampling. We first stratified the high schools based on socioeconomic status of the districts (low, intermediate, and high). Then we randomly selected 8 high schools from each stratum. Finally, subjects were chosen from a registration list (in each selected high school) by simple random sampling to fulfill the sample size requirement (*n* = 300).We did not include participants who reported major depression and anxiety disorder, using of any anti-depressant or sedative medication and who were pursuing a distinct diet, because there is a high probability that people with these disorders and taking antidepressant or sedative medications would have made lifestyle changes like adopting a healthier diet that may bias the results. The study protocol was approved by the research council of the Research Institute for Nutrition and Food Sciences, Shahid Beheshti University of Medical Sciences. All subjects gave written informed consent in accordance with the Declaration of Helsinki (approval number is 054577).

### Assessment of dietary intake

Food consumption was based on a reliable and valid Food Frequency Questionnaire (FFQ) consisting of 168 food items with standard serving sizes typically used in Iran (55). FFQs were collected by specifically trained professional interviewers through private face-to-face interviews. Participants reported their daily, weekly, monthly or yearly of intake frequency for each food item. Daily frequencies for each item were computed. Then, by applying the manual for household measures the daily grams of food intake were calculated (56).

### Anthropometric measurement

Body weight was assessed to the nearest 0.1 kilogram by using digital scales (Seca 881® Germany) while participants were in light clothes with bare feet. Height was evaluated by using a stradiometer in the standing position and was recorded to the nearest 0.1 cm. Body Mass Index (BMI) was computed as weight divided by height squared (kg/m^2^).

### Socio-demographic information

Characteristics including age (years), ethnicity (Fars,Tork, Gilak, others), father/mother job (Unemployed, grade 3 (e.g., laborers), grade 2 (e.g., clerks), grade 1 (e.g., managers and higher), father/mother education (<diploma, diploma, university education), marital status of parents (married, unmarried), salary (USD), chronic disease (yes/no) diet supplement (yes, no), and smoking status (never, previous, current) were collected by a general questionnaire for all subjects.

### Physical activity assessment

Physical activity was assessed using a valid self-reported questionnaire (57) that has been used previously in a sample of Iranian women and demonstrated consistent outcomes (58). Participants were asked to check the activities in which they had participated during the last year. From these reports, the total time spent in particular activities were summed and mean durations were calculated. Total physical activity was expressed as metabolic equivalent-hours per day (Mets/d).

### Other variables

Further information on body image was collected by using 28-items of eating disorder examination questionnaire (EDE-Q-28) (59). The Persian version of EDE-Q-28 which was used in this study (60).

### Dietary inflammatory index (DII®)

The development and validation of the DII are described in detail elsewhere (22). Briefly, developing the DII involved reviewing and scoring nearly 2,000 scientific articles representing cell culture and laboratory animal experiments, and a variety of human studies on diet and six inflammatory markers (i.e., CRP, interleukin (IL)-1b, IL-4, IL-6, IL-10, tumor necrosis factor (TNF)-α). Developing the DII also entailed creation of a world standard database that involved obtaining 11 data sets from around the world to which individuals' intakes of 45 food parameters (consisting of nutrients, spices and whole foods) on which the DII is based, could then be compared.

FFQ-derived dietary data were used to calculate DII scores for all participants. Dietary data were first linked to the previously described regionally representative world database that provided a robust estimate of a mean and standard deviation for each parameter (22). These then became the multipliers to express an individual's exposure relative to the “standard global mean” as a z-score. This score was computed by subtracting the “standard global mean” from the amount reported and dividing this value by the “global standard deviation” of the world population as represented by the 11 data sets used for comparative purposes. To minimize the effect of “right skewing,” this value was then converted to a centered proportion score.

For each individual food parameter, this score was multiplied by the respective food parameter effect score, derived from the literature review, in order to obtain a food parameter-specific DII score (22). All of the food parameter-specific DII scores were then summed to create the overall DII score for each participant in the study, DII = b1^*^n1+b2^*^n2……….b31^*^n31, where b refers to the literature-derived inflammatory effects score for each of the evaluable food parameters and n refers to the food parameter-specific centered percentiles, which were derived from the FFQ-derived dietary data.

For the current study, data on 31 of the 45 DII food parameters could be derived from the FFQ and were thus used for DII calculation. These include: Pro-inflammatory components (energy, carbohydrate, protein, fat, saturated fat, iron, cholesterol, trans-fat, vitamin B12) and anti-inflammatory components (alcohol, fiber, mono-unsaturated fat, poly-unsaturated fat, omega-3, omega-6, niacin, thiamin, riboflavin, magnesium, zinc, vitamin A, vitamin C, vitamin E, vitamin D, vitamin B6, folic acid, beta-carotene, tea, turmeric, garlic, and onions).

### Psychological assessment of depression, anxiety and stress

The Persian version of Depression, Anxiety, Stress Scale-21 (DASS-21) which was introduced by Lovibond and Lovibond (61) has been used to determine the level of depression, anxiety and stress in our sample population. This questionnaire has three subscales and each of them consists of seven items. The score of each subscale is attained by adding the scores of relevant questions. The Persian version of the DASS-21 was found to be a reliable and valid tool to examine the level of depression, anxiety and stress among Iranian adolescents (62).

### Statistical analysis

Study participants' characteristics were described according to two parameters: (1) reporting of at least depressive symptoms and (2) tertiles of the DII. Comparisons were carried out using *t*-tests and ANOVA for continuous variables and chi-square test for categorical variables. Multiple linear and logistic regression analysis were then used to calculate adjusted beta estimates and odds ratios (ORs) and 95% confidence intervals (CIs) for both DASS-21 as continuous and as categorical variable (DASS-21 > 9) in relation to DII in 2 separate models. Model I adjusted for total energy intake and age, model 2 additionally adjusted for physical activity, marital status, income, smoking, BMI, and presence of chronic disease.

## Results

Table 1 describes distribution of characteristics across categories of DASS-21 scores. Females with at least moderate level of depressive symptoms had lower level physical activity and greater dietary supplement use and were either past/current smokers. Table 2 shows distribution of characteristics across tertiles of DII. Females in tertile 3 had higher BMI compared to females in tertile 1. Table 3 describes the results for depression as a continuous score and as a dichotomous outcome where the scores were categorized on having at least a moderate level of depression symptoms (DASS > 9). Significant associations were observed for both types of outcomes for both models. Results are described for the full model, females in the third tertile had significantly higher depression scores (β = 1.67, 95% C.I. 0.04, 3.31); odds of having at least moderate depressive symptoms (DASS-21 > 9) (OR = 3.96, 95% CI 1.12, 13.97) compared to females in tertile 1 (Table 3).

**Table 1:** Characteristics of participants according to different categories of depressive symptoms, Study of Diet-Inflammation and Depression in Iranian Adolescent Females, 2014 to 2015.

**Characteristics^a^^,^^b^**	**Normal symptoms (DASS ≤ 9) *N* = 257**	**At least mild level of depressive symptoms (DASS > 9) *N* = 43**	***P*-value**
Age, (years) (mean ± *sd*)	16.2 ± 1.0	16.3 ± 1.1	0.69
Physical Activity (METs/d)(mean ± *sd*)	36.2 ± 5.7	34.7 ± 5.0	0.11
BMI (kg/m2)(mean ± *sd*)	22.3 ± 4.6	22.3 ± 4.9	0.98
Smoking (%)			0.002
Never	96.9	90.7	
Current/Past	3.1	9.3	
Chronic disease (%)			0.38
Yes	2.3	4.6	
No	97.7	95.4	
Diet supplement use (%)			0.47
Yes	34.0	39.5	
No	66.0	60.5	
Parental marital status (%)			0.20
Yes	95.3	90.7	
No	4.7	8.3	

a *Significance testing was based on t-test for continuous variables*.

b *Chi-square test was used for categorical variables*.

**Table 2:** Participant characteristics by tertiles of dietary inflammatory index (DII)^c^, Study of Diet-Inflammation and Depression in Iranian Adolescent Females, 2014 the 2015.

**Characteristics^a^^,^^b^**	**Tertile 1**	**Tertile 2**	**Tertile 3**	***P*-value**
Age, (years) (mean ± *sd*)	16.1 ± 1.0	16.3 ± 1.0	16.2 ± 0.9	0.83
Physical activity (METs/d)(mean ± *sd*)	36.3 ± 5.9	36.2 ± 5.0	35.4 ± 5.9	0.28
BMI (kg/m2)(mean ± *sd*)	21.4 ± 4.8	22.7 ± 4.4	23.0 ± 4.6	0.02
Smoking (%)				0.39
Never	97.0	97.0	93.0	
Current/Past	3.0	3.0	7.0	
Chronic disease (%)				0.59
Yes	2.0	4.0	2.0	
No	98.0	96.0	98.0	
Diet supplement use (%)				0.17
Yes	42.0	30.3	32.0	
No	58.0	69.0	68.0	
Marital status (%)				0.29
Yes	95.0	97.0	92.0	
No	5.0	3.0	8.0	

a *Significance testing was based on ANOVA for continuous variables*.

b *Chi-square test was used for categorical variables*.

c *Tertile 3 indicates the group with the most pro-inflammatory diet and tertile 1 indicates the group with most anti-inflammatory diet*.

**Table 3:** Beta estimates and odds ratios and confidence intervals for the association between DII as tertiles and depressive symptoms, Study of Diet-Inflammation and Depression in Iranian Adolescent Females, 2014 to 2015.

	**Beta Estimates for depressive symptoms expressed as continuous DASS-21 score**				**Odds Ratios for at least moderate level of depressive symptoms (DASS-21** > **9)**			
**DII^d^**	**Tertile 1**	**Tertile 2**	**Tertile 3**	***P*-trend^c^**	**Tertile 1**	**Tertile 2**	**Tertile 3**	***P*-trend^c^**
DASS-21 > 9/DASS ≤ 9					12/88	17/82	14/87	
Model 1^a^	0	1.47 (0.04, 2.89)	1.97 (0.30, 3.64)	0.03	1	2.70 (1.03, 7.12)	3.01 (0.94, 9.59)	0.09
Model 2^b^	0	1.57 (0.19, 2.94)	1.67 (0.04, 3.31)	0.07	1	3.03 (1.11, 8.26)	3.96 (1.12, 13.97)	0.03

a *Adjusted for age and energy*.

b *Mode 1+physical activity, BMI, smoking, presence of chronic disease, diet supplement use, salary and marital status*.

c *Tests for linear trend were performed by assigning the median value of each category to each participant in that group*.

d *Tertile 3 indicates the group with the most pro-inflammatory diet and tertile 1 indicates the group with most anti-inflammatory diet*.

## Discussion

In this study we report a significant positive association between increasing inflammatory potential of diet and depressive symptoms among adolescent females in Iran. To date, this is the first study to examine this association in adolescent females. A case-control study conducted in Iran showed that a healthy dietary pattern is protective against major depressive disorders, while no such association was observed with the unhealthy dietary patterns. The healthy pattern was characterized by high intakes of fish, poultry, low fat dairy, high fat dairy, coffee, fruits, and nuts, fruit juices, vegetables, legumes, and olives, and low intakes of refined grains, fats, and soft drinks. The unhealthy dietary patterns, on the other hand, were characterized by high intakes of processed meats, red meat, tea, fried potatoes, whole grains, refined grains, snacks, cookies, oils, sugar, and soft drinks (63). Previous reports suggest that men are more likely to have mental health problems than women (64). The current work shows an association between inflammatory potential of diet and depressive symptoms, among the female adolescents. While some of the earlier studies also reported associations between dietary inflammation potential and risk of depression among women; these were female-only cohorts where inflammatory potential of diet was determined by two different methods (inflammatory dietary pattern and DII) (14, 53). Results from the Nurses' Health Study revealed a 30–40% increased risk of depression, among women in the highest quintile compared to women in the lowest quintile of inflammatory dietary pattern (14). Twelve-years' follow-up of middle-aged women in Australia (*n* = 6,438) identified a 20% lower risk of depression among those whose diets were in the highest DII quartile compared to those in the lowest DII quartile (53).

This study is unique because for the first time this association has been examined in a Middle-Eastern population whose dietary habits and culture is very different from the Western population where this relationship has previously been examined (48–53). Traditional Iranian diet consist of food like cooked rice, mixed pilaf, rice, and stew, rice, high protein dishes and stuffed vegetables (65).

In agreement with our findings, the results of the only other study that examined the association between inflammatory potential of diet and depression showed that the hazards ratio for participants in the highest quintile of the DII (strongly pro-inflammatory) was 1.47 (95% confidence interval (CI): 1.17–1.85) compared with those in the bottom quintile, with a significant dose-response relationship (*p* for trend = 0.01) (66).

The DII, thus far, has been shown to be associated with several inflammatory markers and various chronic inflammation related outcomes. For instance, higher DII scores were positively associated with various inflammatory markers, including C-reactive protein (67, 68), interleukin-6 (69, 70), and homocysteine (69). Additionally, the DII has been shown to be associated with bone mineral density among postmenopausal women in Iran (70), two colorectal cancer case-control studies in Spain and Italy (71, 75) and in a cohort study in women in the USA (37, 71), esophageal cancer (72–74), breast cancer (75), pancreatic cancer (76), prostate cancer (77, 78), cardiovascular diseases (79, 80) and biomarker of aging (81).

The direct association between the DII and the odds of depression observed in this study could be explained by the fact many of the anti-inflammatory components of the index, namely zinc, omega 3 fatty acids, and coffee, have been shown to be negatively associated with the risk of depression (13). On the other hand, pro-inflammatory components of the index, such as energy and carbohydrate, have been linked to a higher risk of depression. For instance, results from a large cohort study conducted in the USA showed that frequent consumption of sweetened beverages, especially diet drinks, increases the risk of depression among older adults, whereas coffee consumption lowered the risk (11). Various studies have been conducted examining the association between dietary pattern and depression. In a systematic review conducted on 21 studies high intakes of fruit, vegetables, fish, and whole grains may be associated with a reduced depression risk (82). There is substantial evidence linking inflammation and depression (83–86).

Despite its strengths, our study had some limitations, which should also be considered in interpreting the results. First, the validity and reliability of FFQ has not been established in the context measuring dimensions of depression measured using DASS-21. Second, we could not directly infer causality due to the cross-sectional nature of the study design. Other limitations include small sample size, and the possibility of recall bias. Fourth, in this study, data were available on 31 of the 45 food parameters; absence of information on the remaining food parameters can be considered as a limitation. Additionally, validity and reliability of FFQ has not been established in the context measuring dimensions of depression measured using DASS-21. Another limitation is the inability to evaluate the history of stressful life events in the last 12 months. Self-reported dietary methods like the FFQ is subjected to bias of under reporting and implausible values for energy intake, the inability to measure this bias and adjust for it in the analyses is a limitation.

In conclusion, female adolescents with a pro-inflammatory diet have greater odds of having at least a moderate level of depressive symptoms. So, promoting diets with a higher concentration of anti-inflammatory foods, such as vegetables and fruits, at younger age may be protective against the development of depression. However, further studies analyzing the link between diet, inflammation and depression are warranted among both men and women to further elucidate the role of diet in the development of depression and other mental disorders.

## Author contributions

NS calculated the DII, ran the analyses and also wrote the first draft of the manuscript. Upon receiving comments from the co-authors he also made changes to the manuscript and finalized it. JRH, AN, BB, FN, and BR reviewed and provided important input to the paper.

### Conflict of interest statement

JRH owns controlling interest in Connecting Health Innovations LLC (CHI), a company planning to license the right to his invention of the dietary inflammatory index (DII) from the University of South Carolina in order to develop computer and smart phone applications for patient counseling and dietary intervention in clinical settings. NS is an employee of CHI. The remaining authors declare that the research was conducted in the absence of any commercial or financial relationships that could be construed as a potential conflict of interest. The handling Editor declared a past co-authorship with the authors NS, JRH.
